# ADP-Ribose Activates the TRPM2 Channel from the Sea Anemone *Nematostella vectensis* Independently of the NUDT9H Domain

**DOI:** 10.1371/journal.pone.0158060

**Published:** 2016-06-22

**Authors:** Frank J. P. Kühn, Cornelia Kühn, Mathis Winking, Daniel C. Hoffmann, Andreas Lückhoff

**Affiliations:** Institute of Physiology, Medical Faculty, RWTH Aachen, D-52057 Aachen, Germany; University of South Florida, UNITED STATES

## Abstract

The human redox-sensitive Transient receptor potential melastatin type 2 (*h*TRPM2) channel contains the C-terminal Nudix hydrolase domain NUDT9H which most likely binds ADP-ribose. During oxidative stress, the intracellular release of ADP-ribose triggers the activation of *h*TRPM2. The TRPM2 orthologue from *Nematostella vectensis* (*nv*) is also stimulated by ADP-ribose but not by the oxidant hydrogen peroxide. For further clarification of the structure-function relationships of these two distantly related channel orthologues, we performed whole-cell as well as single channel patch-clamp recordings, Ca^2+^-imaging and Western blot analysis after heterologous expression of wild-type and mutated channels in HEK-293 cells. We demonstrate that the removal of the entire NUDT9H domain does not disturb the response of *nv*TRPM2 to ADP-ribose. The deletion, however, created channels that were activated by hydrogen peroxide, as did mutations within the NUDT9H domain of *nv*TRPM2 that presumably suppress its enzymatic function. The same findings were obtained with the *nv*TRPM2 channel when the NUDT9H domain was replaced by the corresponding sequences of the original *h*NUDT9 enzyme. Whenever the enzyme domain was mutated to presumably inactive variants, channel activation by hydrogen peroxide could be achieved. Moreover, we found strong evidences for ADPRase activity of the isolated NUDT9H domain of *nv*TRPM2 in co-expression experiments with the C-terminally truncated *nv*TRPM2 channel. Thus, there is a clear correlation between the loss of enzymatic activity and the capability of *nv*TRPM2 to respond to oxidative stress. In striking contrast, the channel function of the *h*TRPM2 orthologue, in particular its sensitivity to ADP-ribose, was abrogated by already small changes of the NUDT9H domain. These findings establish *nv*TRPM2 as a channel gated by ADP-ribose through a novel mechanism. We conclude that the endogenous NUDT9H domain does not directly affect ADP-ribose-dependent gating of the *nv*TRPM2 channel; instead it exerts an independent catalytic function which possibly controls the intracellular availability of ADP-ribose.

## Introduction

The human Transient receptor potential melastatin-related 2 (*h*TRPM2) channel is a Ca^2+^ permeable cation channel which has been described as celluar redox sensor [1]. There is accumulating evidence for a pivotal role of *h*TRPM2 in numerous metabolic and neuronal diseases such as diabetes mellitus, bipolar disorder and Alzheimers’s disease (e.g. reviewed in [2–4]).

Currently our knowledge of the gating mechanisms of this complex protein is still very limited. A promising experimental approach for further investigations is the systematic analysis of species variants of TRPM2 [5].

The family of TRP channels represents a phylogenetically ancient group of ion channels. This is for example evidenced by the discovery of TRPM2-like orthologues in unicellular protists as well as in basal metazoans e.g. the sea anemone *Nematostella vectensis* [6;7]. Thus, *nv*TRPM2 and *h*TRPM2 have undergone an independent phylogenetic development since the divergence of cnidarians and bilaterians about 700 million years ago [8]. Although the TRPM orthologue of *N*. *vectensis* shares distinct similarities with the S1-S2 transmembrane linker of the human TRPM3 channel [5], it should be indicated as TRPM2 because its C-terminus contains the characteristic and functional decisive structural element of the *h*TRPM2 channel, the NUDT9 homology (NUDT9H) domain [9;10]. This domain, with striking homology to the human adenosine 5’-diphosphoribose (ADPR) pyrophosphatase NUDT9 of the Nudix hydrolase family [11] controls the activation of *h*TRPM2 by intracellular ADPR [9;12]. The TRPM2 channel of *N*. *vectensis* (*nv*TRPM2) has been recently characterized in our lab and exhibited currents in response to ADPR as well, with even greater sensitivity and faster kinetics than its human counterpart. However, H_2_O_2_ which is an established further activator of *h*TRPM2 [1,13], was completely without effect on *nv*TRPM2 [5].

The NUDT9H domain of *h*TRPM2 is believed to bind ADPR without enzymatic function as a prerequisite for channel activation [14–17]. Studies on the NUDT9 enzyme have revealed that the sequence motif, glutamate-phenylalanine (EF), within the catalytic active site is critical for ADPR pyrophosphatase activity [10]. Exactly these two residues are changed to isoleucine-leucine (IL) in the NUDT9H domain of the *h*TRPM2 channel. The opposite mutation of EF to IL in the enzyme reduces catalytic activity to 1% [14], whereas reconstitution of IL to EF in *h*TRPM2 completely abolishes any channel function [15;16].

Interestingly, the NUDT9H domain in *nv*TRPM2 contains not the sequence IL but EF at this critical position. Hence, it should be enzymatically active. Moreover, replacing the NUDT9H domain of *nv*TRPM2 with the human version did not affect channel responses to ADPR but surprisingly created additional sensitivity to H_2_O_2_ which is absent in wild-type *nv*TRPM2 [5]. In cells transfected with chimeric *nv*TRPM2 channels containing the human version of the NUDT9H domain, extracellular application of H_2_O_2_ produced large but transient currents after a characteristic delay. Several specific sequence adjustments of the NUDT9H domain of *nv*TRPM2 according to the human template produced the same principal result, i.e. the sensitivity to ADPR remained unaltered and sensitivity to H_2_O_2_ was gained [5].

These findings along with a sequence comparison of the corresponding NUDT9 motifs in man and sea anemone may cast doubt on an essential role of the NUDT9H domain for ADPR gating in *nv*TRPM2. The aim of the present study, therefore, was a rigorous test for the hypothesis that NUDT9H is not required for stimulation of channel activity by ADPR in *nv*TRPM2. Moreover, we wished to find out the importance of an enzymatic active NUDT9H domain for the activation by H_2_O_2_. We report that loss of NUDT9H from *nv*TRPM2 creates a channel with almost fully preserved sensitivity for ADPR and newly gained sensitivity for H_2_O_2_. The latter is strongly correlated with the loss of function of the endogenous enzyme domain that, in its intact form, most probably prevents intracellular accumulation of ADPR. Thus, the NUDT9H domain of TRPM2 orthologues obtained from two far distantly related species have experienced completely different adaptations after the phylogenetic separation of both species.

## Material and Methods

### Molecular cloning

Subcloning of the TRPM2 cDNA from human and from *N*. *vectensis* (*nv*TRPM2; jgi.Nemve1.248535|estExt_fgenesh1_pg.C_6220005) into the modified pIRES-hrGFP-2a vector (Stratagene, La Jolla, CA, USA) was described previously [5]. The amino acid sequence of *nv*TRPM2 was retrieved from the genomic database JGI (http://www.jgi.doe.gov/). The corresponding cDNA was synthesized by MWG-Biotech (Ebersberg, Germany). The codon usage was adapted during synthesis to ensure optimal expression in HEK-293 cells and the DNA sequence was verified by double-strand DNA sequencing by MWG-Biotech. The cDNA from the human NUDT9 enzyme (Accession No: NM_024047.3) was purchased from AMS Biotechnology, (Abingdon, UK) and subcloned into the modified pIRES-hrGFP2a vector via an *Eco* RI + *Xba* I cloning step. For the co-expression expriments with NUDT9 enzyme variants, the EGFP open reading frame of the pIRES-hrGFP-2a vector was optionally replaced by a DsRed open reading frame. Therefore, it was possible to detect positive co-expression of TRPM2 channels (green fluorescence) and NUDT9 enzymes (red fluorescence) in a single cell. In addition to the wild-type *h*NUDT9 enzyme the following two enzyme variants were used for co-expression studies: The amino acid residues (aa) 77–350 of wild-type *h*NUDT9 enzyme were either replaced by the corresponding NUDT9H sequences of the *h*TRPM2 channel (aa 1253–1503) or the *nv*TRPM2 channel (aa 1289–1551). Site-directed mutagenesis was performed using the QuikChange mutagenesis system (Stratagene, La Jolla, CA, USA). Defined oligonucleotides were obtained from MWG-Biotech. Each point mutation and chimeric channel construct was verified by DNA sequencing (MWG-Biotech). For the deletion of the NUDT9H domain, C-terminally truncated TRPM2 variants were generated by the introduction of stop codons at amino acid position 1167 (*h*TRPM2) and 1208 (*nv*TRPM2). Optionally, the full-length TRPM2 channels as well as the truncated channel variants were C-terminally fused with a triple hemagglutinin (3xHA)-tag for easy detection in Western blot analysis. This was performed as follows: The genuine pIRES-hrGFP-2a expression vector already includes a 3xHA-tag downstream of the multiple cloning site followed by two consecutive stop codons. The single *Xho* I site in front of the 3xHA-tag was changed to a unique *Afl* II site. The full-length as well as the truncated channel variants were also modified by conversion of the corresponding stop codon of the open reading frame into a unique *Afl* II site. Subsequently, the modified vector fragment containing the 3xHA-tag in proper reading frame was added to each of the prepared expression vector-channel constructs via an *Afl* II + *Sca* I cloning step. For some experiments, the original 3xHA-tag of the pIRES-hrGFP-2a vector/TRPM2 channel-constructs was changed into a single (1x) HA-tag. This was achieved by introducing a stop codon immediately downstream of the first HA-tag unit. All procedures were performed in accordance to the respective manufacturer’s instructions, unless indicated otherwise.

### Cell culture and transfection

HEK-293 cells were obtained from the German Collection of Microorganisms and Cell Cultures (Braunschweig, Germany) and cultured in DMEM media (Biochrome, Berlin, Germany) supplemented with 4 mM L-glutamine and 10% (v/v) fetal calf serum (Biochrome) and 2 mM sodium pyruvate. Transient transfections of HEK-293 cells with the cDNAs of various *h*TRPM2 and *nv*TRPM2 variants were performed using the FuGene 6 transfection reagent (Roche, Mannheim, Germany) according to the manufacturer’s instructions. As controls, cells were transfected with the pIRES-hrGFP-2a vector alone. The transfected cells were maintained for 24 h in an incubator at 37°C and 5% CO_2_. Subsequently, the cells were seeded on poly-lysine-coated glass coverslips at a suitable dilution and further incubated for 3–4 h. Then, patch-clamp and calcium imaging experiments were carried out with cells visibly positive for EGFP (and additionally DsRed in co-expression experiments). At least three independent transfections were used for each experimental group.

### Cell surface biotinylation and Western blot analysis

Biotinylation assays were performed with the Pierce Cell Surface Protein Isolation Kit according to the manufacturer’s instructions (ThermoFisher Scientific, USA). In brief, transfected, sub-confluent HEK-293 cells (90%) were biotinylated and lysed. Samples (600 μg) were incubated with NeutrAvidin beads and a small aliquot of total cell lysate was used as input control. Elution was performed with SDS sample buffer and subjected to SDS-PAGE and Western blot analysis. Detection of β-actin with mouse-anti-β-actin antibody (1:2000; Sigma-Aldrich, USA) and rabbit-anti-mouse-HRP conjugated secondary antibody (1:2000; DAKO A/S, Agilent, USA) was used to rule out that cytosolic proteins in damaged cells were biotinylated. Alternatively, the membrane fractions of transfected HEK-293 cells were prepared by differential ultracentrifugation according to the protocol of Vriens and coworkers [18], as described previously [19]. Normalized samples were subjected to reducing SDS-PAGE (4–12% Bis-Tris, NuPAGE, Novex; ThermoFisher Scientific) and Western blot analysis. Expression was determined using a primary monoclonal mouse-anti-HA antibody (1:2000; Sigma-Aldrich) and a rabbit-anti-mouse-HRP conjugated secondary antibody (1:2000; DAKO A/S). The endogenous expression of human NUDT9 enzyme in HEK-293 cells was tested with Western blot analysis of total cell lysate using a primary monoclonal mouse-anti-*h*NUDT9 antibody (OriGene Technologies, Rockville, USA) and a rabbit-anti-mouse-HRP conjugated secondary antibody (1:2000; DAKO A/S). Detection was accomplished using the enhanced chemiluminescence detection system (ECL, Amersham Bioscience, USA).

### Electrophysiology

Whole-cell recordings were performed using an EPC 9 amplifier equipped with a personal computer with Pulse 8.5 and X Chart software (HEKA, Lamprecht, Germany). The standard bath solution contained (in mM) 140 NaCl, 1.2 MgCl_2_, 1.2 CaCl_2_, 5 KCl, 10 HEPES, pH 7.4 (NaOH). For Na^+^ free solutions, Na^+^ was replaced by 150 mM N-methyl-D-glucamine (NMDG) and the titration was performed with HCl. The pipette solution contained (in mM) 145 CsCl, 8 NaCl, 2 MgCl_2_, 10 HEPES, pH 7.2 (CsOH). The Ca^2+^ concentration of the pipette solution was adjusted to 1 μM (0.886 mM Ca^2+^, 1 mM Cs-EGTA) as calculated using the *MAXC*-program: (http://www.stanford.edu/~cpatton/maxc.html). For the stimulation of TRPM2 channels, Adenosine diphosphate ribose (ADPR; 100 mM stock solution in distilled water) or Adenosine diphosphate ribose-2’-phosphate (ADPRP; 5 mM stock in pipette solution) was added to the pipette solution yielding final concentrations between 0.05 and 1.2 mM (ADPR) and 0.005 and 0.5 mM (ADPRP). Alternatively, TRPM2 currents were evoked by superfusion of the cells with standard bath solution containing 10 mM H_2_O_2_ (diluted from a 30% stock solution). Unless stated otherwise, the experiments were performed at room temperature (21°C) and the current-voltage relations were obtained during voltage ramps from –150 to +150 mV and back to -150 mV applied over 200 ms. The holding potential was -60 mV. For the analysis the maximal current amplitudes (pA) in a cell were divided by the cell capacitance (pF), a measure of the cell surface. The result is the current density (pA/pF).

Single-channel currents were recorded from inside-out patches at room temperature (21°C). Patch pipettes were made of borosilicate glass (Hilgenberg, Malsfeld), coated with dental wax (Moyco, Philadelphia, USA), and had tip resistances between 5 and 7 MΩ. Recordings were made with an Axopatch 200B amplifier in combination with a Digidata 1440A AD/DA converter controlled by the pCLAMP10 software suite (Axon Instruments, Foster City, CA). A gap-free acquisition mode was used with analogous filtering at 5 kHz performed with 4-pole Bessel filter (3 dB). External (pipette) and internal (bath) solutions were the same as those in whole-cell recordings. Patches with *h*TRPM2 and *nv*TRPM2 variants were activated by addition of ADPR (0.4 mM) to the bath (internal) solution. Analysis was performed after additional filtering (1 kHz) as previously described [20].

### Calcium imaging experiments

For fluorescence imaging of (Ca^2+^)_i_ HEK-293 cells on poly-lysine-coated glass coverslips were loaded in standard bath solution containing membrane-permeable Fura-2 acetoxymethyl ester (1.5 ng/μl; ThermoFisher Scientific, USA) and pluronic acid (0,025%) for 20 min at 37°C. Fluorescence was alternately excited at 340 nm and 380 nm using the Polychrome IV monochromator (TILL Photonics, Germany). The emitted fluorescence was measured at 510 nm using a Sensicam (IMAGO). Fluorescence was corrected for background at each wavelength. Measurements were obtained at room temperature (21°C). The standard bath solution and stimulation with H_2_O_2_ were identical to those described for the patch-clamp experiments.

### Data analysis and statistics

Data are expressed as the mean ± s.e.m. The comparison of two groups was performed using an unpaired Student's t-test. Calcium imaging experiments were statistically evaluated using a one-way ANOVA and the Bonferroni correction was applied when multiple comparisons were performed with the same control data. Differences were considered significant at * P < 0.5, ** P < 0.01 and *** P < 0.001.

## Results

### Corresponding mutations within the NUDT9H domain cause loss of function in *h*TRPM2 but gain of function (activation by hydrogen peroxide) in *nv*TRPM2

**Fig 1:**
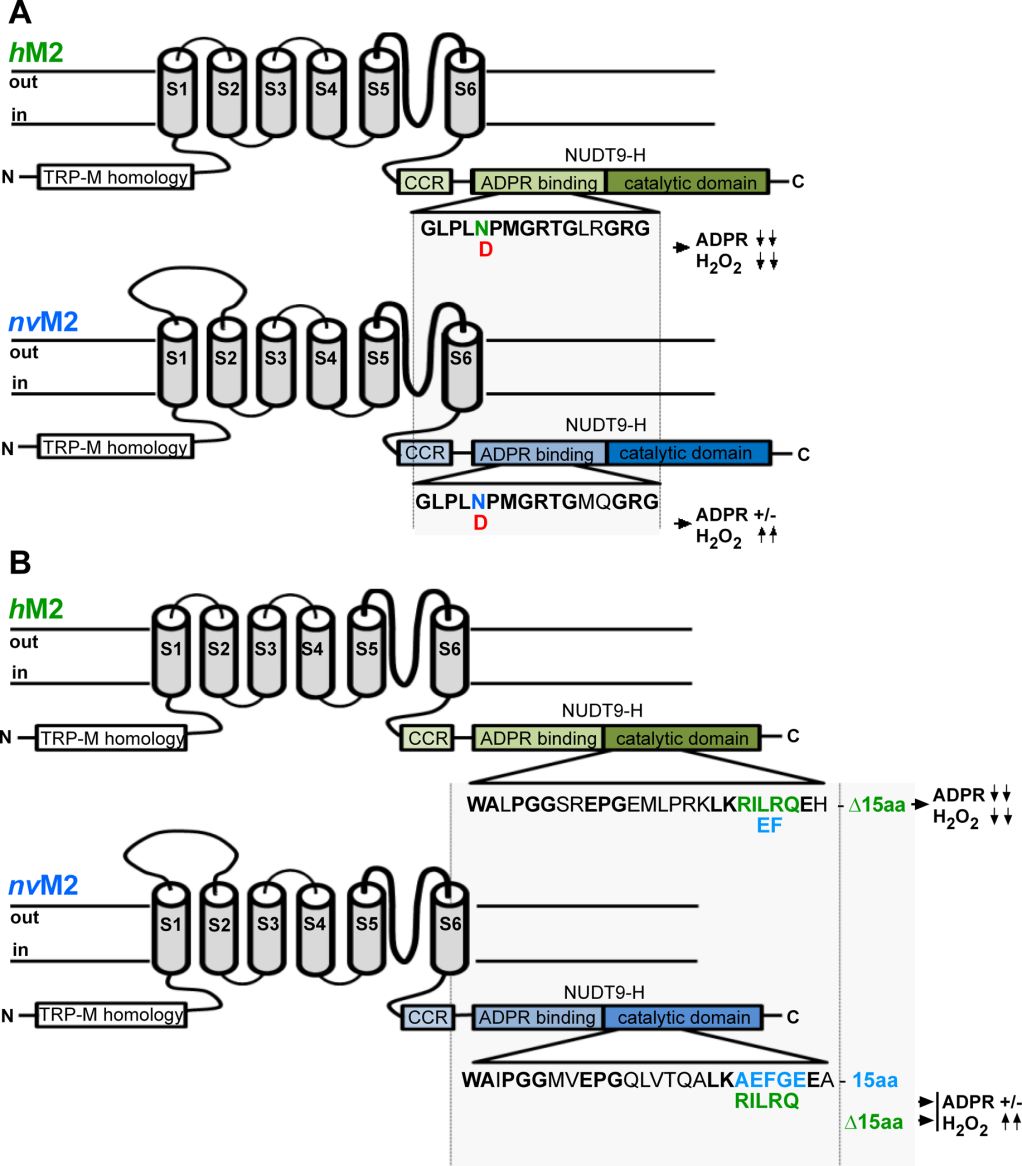
Summary of NUDT9H mutations of *h*TRPM2 and *nv*TRPM2 and their effects on channel function. Characteristic TRPM2 regions like the N-terminal TRPM homology region, the six transmembrane segments, as well as the C-terminal coiled-coiled region and the NUDT9H domain are indicated. The NUDT9H domain is divided into the putative N-terminal ADPR binding region and the C-terminal catalytic domain. **(A)** Human TRPM2 (green) with the mutation N1326D in the ADPR binding region which abolishes both the sensitivity to ADPR and to H_2_O_2_ (symbolized with **↓↓**). In contrast, the corresponding mutation N1365D in *nv*TRPM2 (blue) does not change the sensitivity to ADPR (+/-) but produces strong sensitivity to H_2_O_2_ (**↑↑**). **(B)** Human TRPM2 in which the double mutation IL to EF within the catalytic site abolishes both the sensitivity to ADPR and to H_2_O_2_. The deletion of 15 amino acid residues immediately downstream of the catalytic site which is characteristic for *h*TRPM2 is also indicated. In contrast, the reciprocal mutations in the catalytic site of *nv*TRPM2 (EF to IL or the deletion Δ15) again do not change the sensitivity to ADPR but produce sensitivity to H_2_O_2_.

The principal differences between *h*TRPM2 and *nv*TRPM2 are illustrated in **Fig 1**.

**Fig 2:**
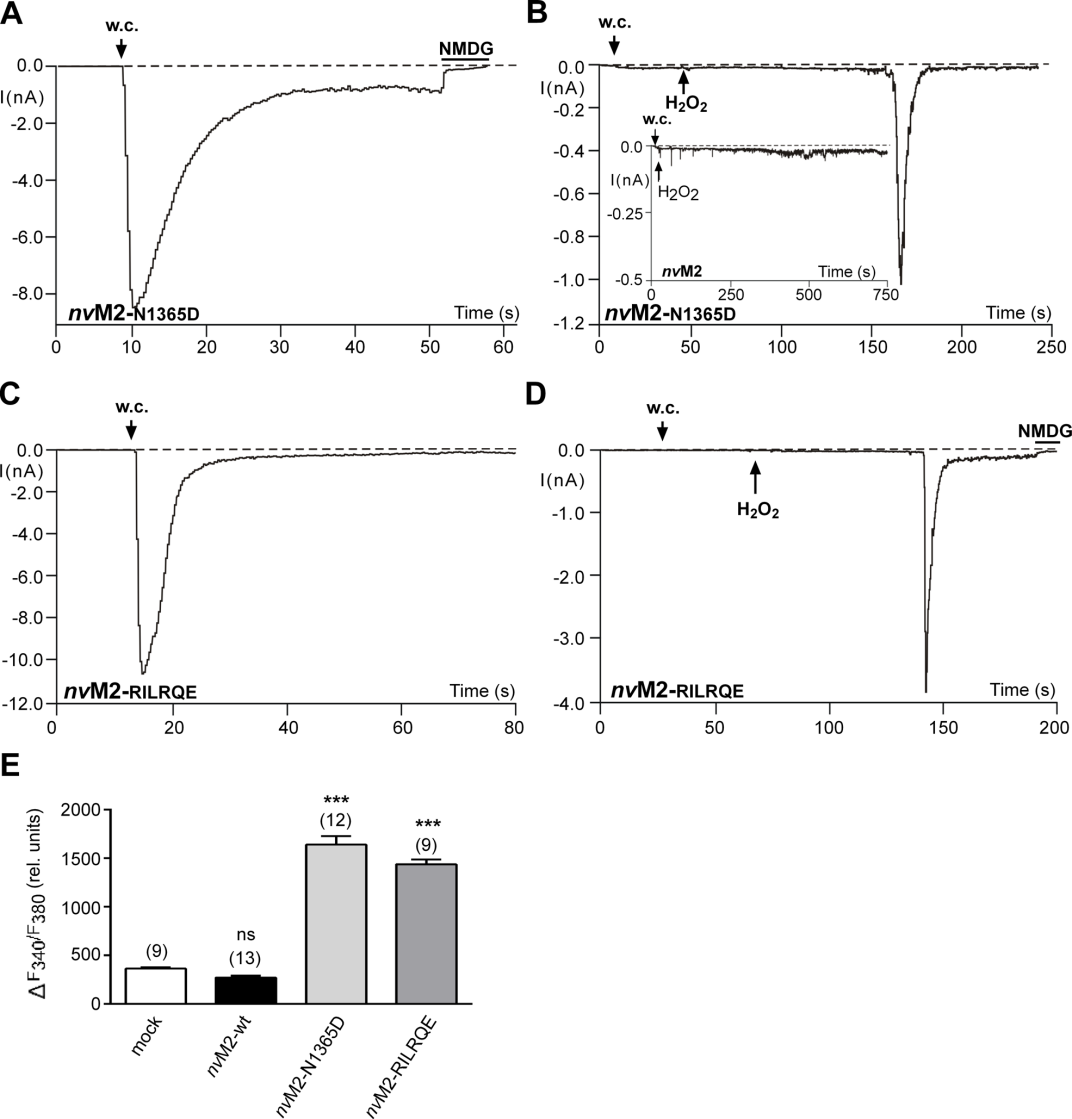
Functional characterisation of *nv*TRPM2 variants with mutations in the NUDT9H region. **(A-D)** Representative whole cell patch-clamp experiments. The variants were *nv*TRPM2-N1326D **(A and B)** and *nv*TRPM2-RILRQE **(C and D)**. Stimulation of currents was performed either with ADPR (50–100 μM) in the pipette **(A and C)** or with H_2_O_2_ (10 mM) applied to the bath at the time point indicated by an arrow **(B and D)**. As negative control for the stimulation with H_2_O_2_, a recording of wild-type *nv*TRPM2 is included in **panel B (inset)**. The intracellular Ca^2+^ concentration was adjusted to 1 μM. Current flow was inhibited by superfusion of the cells with a solution containing NMDG as main cation (horizontal bars). For each variant similar results were obtained from a least 3 independent experiments. **(E)** Summary of calcium imaging experiments. Maximal increases in (Ca^2+^)i, as indicated by an increased F340/F380 ratio, were evoked by extracellular H_2_O_2_ (10 mM). The variants (see above) were compared with mock-transfected cells as well as with cells transfected with wild-type *nv*TRPM2. *** indicates a significant difference (P < 0.001) evaluated with a one-way ANOVA and the Bonferroni correction. (n = 9–13). Error bars are s.e.

In *h*TRPM2, the C-terminal NUDT9H domain putatively contains an ADPR-binding pocket, followed by the catalytic site [14]. Specific changes in either of these two regions lead to a complete loss of channel function, affecting both, responses to ADPR and to H_2_O_2_. A striking example [15] is the point mutation N1326D in the putative binding site (**Fig 1A**). In strong contrast, *nv*TRPM2 with the corresponding mutation N1365D characterized in the present study (**Fig 1A**) exhibited currents in response to ADPR (**Fig 2A**) virtually identical to the currents of wild-type *nv*TRPM2 [5]. Thus, the mutation deleterious to *h*TRPM2 activity is without effect on *nv*TRPM2. Moreover, extracellular application of H_2_O_2_ elicited a characteristic and delayed current response in *nv*TRPM2-N1365D (**Fig 2B**) that was never observed in wild-type controls (**Fig 2B inset**; n = 8; also see ref. 5). Hence, the sensitivity for H_2_O_2_ was created by this specific mutation. Almost identical results were obtained by a mutation within the catalytic site (**Fig 1B**). For *h*TRPM2, the sequence 1402-KRILRQ-1409 is essential, as a substitution of the motif IL with EF is deleterious [15;16]. In wild-type *nv*TRPM2, the corresponding sequence is 1440-KAEFGE-1447, far more closely resembling the human NUDT9 enzyme (with the almost identical sequence 227-KREFGE-234) than the human TRPM2 channel. Substitution of the *nv*TRPM2 sequence motif KAEFGE with the human counterpart KRILRQ again left responses to ADPR unaltered (**Fig 2C**) and produced sensitivity for H_2_O_2_ (**Fig 2D**). The electrophysiological results were confirmed in calcium imaging studies where extracellular H_2_O_2_ induced large increases in (Ca^2+^)i in cells transfected with either *nv*TRPM2-N1365D or *nv*TRPM2-RILRQE. In control cells, either mock-transfected or transfected with wild-type *nv*TRPM2 no such increases in (Ca^2+^)i were measured (**Fig 2E**). Increasing the extracellular Ca^2+^ concentration in the absence of a stimulus did not increase (Ca^2+^)i in cells transfected with wild-type *nv*TRPM2 (n = 27), demonstrating that the channels are not constitutively open. Extracellular ADPR was without effect as well (n = 20).

### ADPR-dependent gating of *nv*TRPM2 is independent of the NUDT9 domain

**Fig 3:**
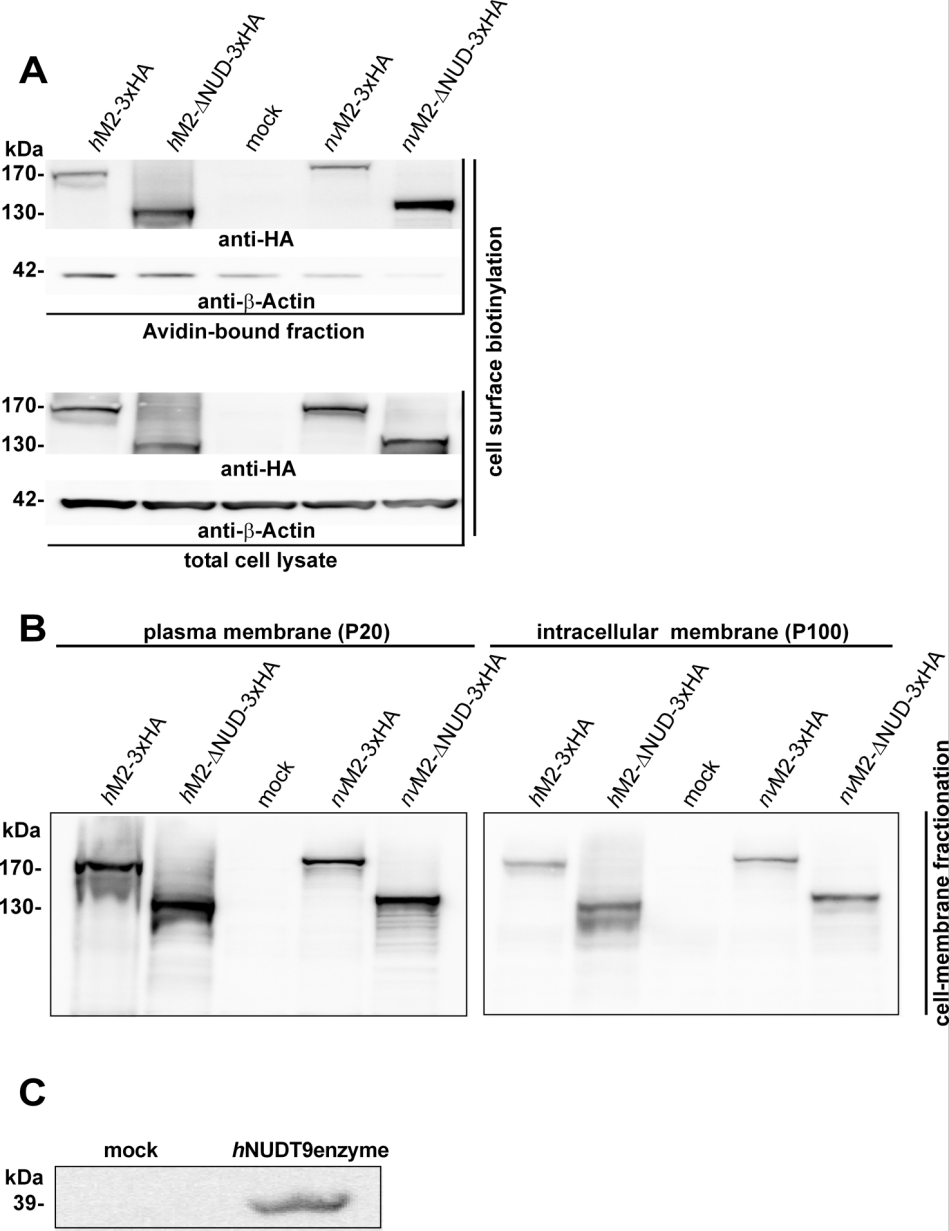
Western blot of surface expressed variants of *h*TRPM2 and *nv*TRPM2 and endogenous NUDT9 expression. **(A)** Cell surface expression, assessed with biotinylation assays, of full-length and truncated channel variants (as indicated), each containing a C-terminally attached 3xHA tag. Western blots on the NeutrAvidin-bound fractions (top) and on total HEK-293 cell lysates (bottom) were probed with anti-HA antibody. Reduced β-actin staining in the Avidin-bound faction rules out biotinylation of cytosolic proteins in damaged cells. **(B)** Western blots on enriched plasma membrane (left) and intracellular membrane fractions (right) of HEK-293 cells expressing the indicated TRPM2 variants. Membrane fractions were prepared with the differential centrifugation method [18] and probed with anti-HA antibody. Mock-transfected cells were used as negative control. Two independent experiments gave similar results. **(C)** Western blot on total HEK-293 cell lysates of mock-transfected cells and cells transfected with cDNA of the human NUDT9 enzyme (as indicated) probed with monoclonal mouse anti-*h*NUDT9 antibody. For each of the Western blots at least two independent experiments were performed to give similar results.

So far, the results indicate that *nv*TRPM2 does not seem to have specific structural requirements for its NUDT9H domain, at least in response to ADPR. Moreover, responses to H_2_O_2_ (absent in wild-type *nv*TRPM2) are created by several mutations within different parts of the NUDT9H region, and all the mutated residues are critical for both, channel function in *h*TRPM2 [15;16] and catalytic function in the human NUDT9 enzyme [10;14]. In this confusing situation, we studied the channel variants *h*TRPM2-ΔNUD and *nv*TRPM2-ΔNUD in which the NUDT9H domains were completely removed (see also Materials and Methods section). To test the successful expression of full-length and truncated channel variants by Western blot analysis, we decided to attach a triple hemagglutinin (3xHA)-tag to the C-termini of each of the constructs (see also Materials and Methods section). The correct insertion of the various channel constructs into the plasma membrane was verified by a cell surface biotinylation assay (**Fig 3A**) and confirmed by an independent method (**Fig 3B**) using differential ultracentrifugation of cell extracts [18], followed by Western blot analysis directed against the HA-tag. As expected, all full-length channel proteins as well as truncated variants were detected with molecular weights as calculated (*h*TRPM2-3xHA 175 kDa, lane1; *h*TRPM2-ΔNUD-3xHA 137 kDa, lane 2; *nv*TRPM2-3xHA 179 kDa, lane 4; *nv*TRPM2- Δ NUD-3xHA 141 kDa, lane 5) in the Avidin-bound fraction representing the pool of biotinylated surface expressed proteins. Thus, all TRPM2 channels studied are reasonably expressed in the cell membrane of HEK-293 cells, notably even the human and Nematostella variants in which the NUDT9H domain is lacking (**Fig 3A and 3B**).). Moreover, we checked the endogenous expression of the human NUDT9 enzyme in HEK-293 cells. As positive control, we used HEK-293 cells transiently transfected with the cDNA of the human NUDT9 enzyme. The Western Blot in **Fig 3C** confirmed the heterologous expression of human NUDT9 in the positive control, whereas native mock-transfected HEK-293 cells did not show expression of NUDT9 enzyme. Therefore any trans-activation of C-terminally truncated TRPM2 channels by endogenously expressed human NUDT9 enzyme is unlikely.

**Fig 4:**
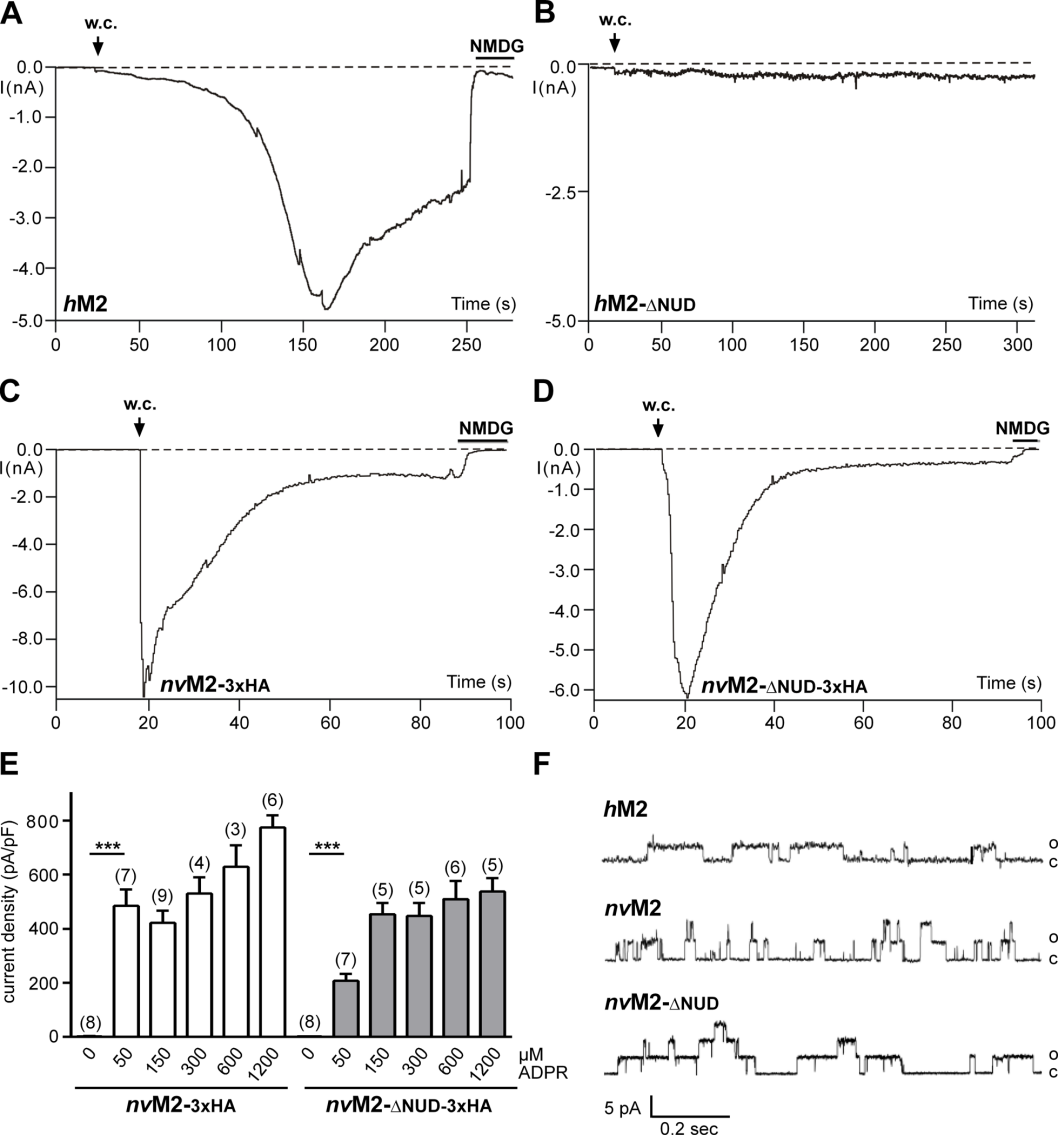
Functional characterisation of TRPM2 variants with the NUDT9H domain removed. **(A)** Control patch-clamp experiment performed on *h*TRPM2 stimulated with ADPR (0.3 mM) and Ca^2+^ (< 10 nM) in the pipette solution. Note the typical slow onset of the current and block by NMDG. **(B)** Absent response with ADPR (0.6 mM) and Ca^2+^ (1 μM) in the pipette solution in *h*TRPM2 lacking the NUDT9H domain. For both *h*TRPM2- Δ NUD variants (with or without 3xHA-tag) similar results were obtained from a least 10 independent experiments. **(C)** Characteristic current development induced by ADPR (0.6 mM) in *nv*TRPM2-3xHA. **(D)** Current response to ADPR (0.1 mM) in the *nv*TRPM2 variant in which the NUDT9 domain is lacking (in both *nv*TRPM2- Δ NUD variants the same results were obtained with or without 3xHA-tag). The intracellular Ca^2+^ concentration was adjusted to 1 μM. **(E)** Relation of current densities to ADPR concentration in cells transfected either with *nv*TRPM2-3xHA or with *nv*TRPM2- Δ NUD-3xHA. Already the smallest concentration of ADPR evoked significant currents (*** P < 0.001; Student's t-test, n = 8) in comparison to the absence of a stimulus. Error bars are s.e. (**F**) Direct activation of inside-out patches by ADPR. The three traces were from TRPM2 channel variants as indicated and obtained rapidly after establishing the inside-out configuration, with ADPR (0.4 mM) already present in the bath. Multiple channels were present in all three patches but the initial activation showed preferentially openings of one or of few channels. The transmembrane potential was +60 mV, chosen because inactivation of *nv*TRPM2 was slower when currents were in the outside direction. Similar results were obtained from at least 4 independent experiments.

The functional characterisation of full-length *nv*TRPM2-3xHA and *nv*TRPM2-ΔNUD-3xHA is shown in **Fig 4**. As control, we used wild-type *h*TRPM2 and *h*TRPM2-ΔNUD. While wild-type *h*TRPM2 exhibited the well-known currents after infusion with ADPR (**Fig 4A**), such currents were completely absent in *h*TRPM2-ΔNUD (**Fig 4B**; n = 12). It should be noted that *h*TRPM2 variants with partial as well as with entire deletion of the NUDT9H domain had already been tested as functionally negative [1;15;16]. The corresponding *N*. *vectensis* variant *nv*TRPM2-ΔNUD-3xHA, however, showed large currents evoked by ADPR. The current onset, in comparison to wild-type *nv*TRPM2-3xHA (**Fig 4C**), may be marginally slower (**Fig 4D**), maximal current amplitudes may be slightly smaller and the concentration-effect curve may be minimally shifted to the left (**Fig 4E**). Unequivocally, however, the results demonstrate ADPR-induced currents in the absence of the NUDT9H domain in *nv*TRPM2-ΔNUD-3xHA. Virtually identical findings were obtained in the two corresponding *nv*TRPM2 variants without C-terminally fused 3xHA-tags (n = 6–9).

### ADPR-dependent activation of full-length and truncated *nv*TRPM2 variants in inside-out patches

To add more experimental proof for a direct activation of *nv*TRPM2 and of *nv*TRPM2-ΔNUD by ADPR, stimulation of the channel was performed in inside-out patches. Immediately after contact of the cytosolic side with ADPR, channel activity was induced (**Fig 4F**) that lasted for a least a minute. Single channel properties of *h*TRPM2 exhibit many unusual characteristics, notably long mean open times in the range of several hundred milliseconds, with very little flickering [9, 12, 20]. It may be assumed that these properties are related to the unique gating mechanisms of *h*TRPM2 that essentially involve the NUDT9H domain. However, similar kinetic properties as of *h*TRPM2 were observed for wild-type *nv*TRPM2 (**Fig 4F**) after activation with ADPR. The slope conductance was 65 pS (*nv*TRPM2) and 75 pS (*nv*TRPM2-ΔNUD), respectively which is also in good agreement with the values of *h*TRPM2 (60–74 pS; e.g. ref. [9, 20, 21]). Moreover, no obvious differences were seen between wild type *nv*TRPM2 and *nv*TRPM2-ΔNUD (**Fig 4F**). These results add to the compelling evidence from whole cell experiments that ADPR directly gates *nv*TRPM2 independently of the NUDT9H domain.

### ADPR-2’-phosphate acts on *nv*TRPM2 in the same way as ADPR

**Fig 5:**
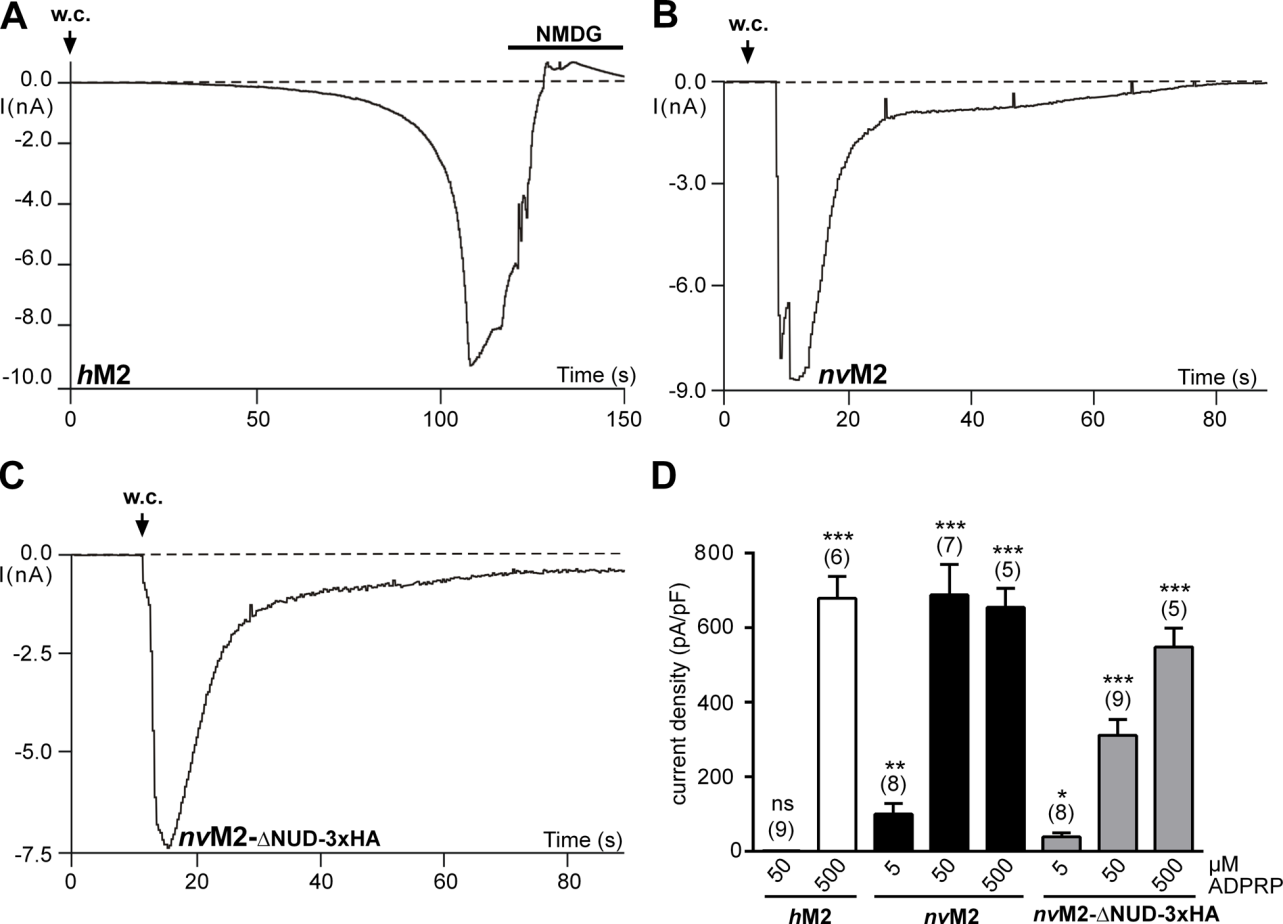
Effects of ADPR-2’-phosphate (ADPRP) on channel variants of *h*TRPM2 and *nv*TRPM2. **(A-C)** Representative patch-clamp experiments either on wild-type *h*TRPM2, wild-type *nv*TRPM2 or *nv*TRPM2- Δ NUD-3xHA as indicated. Stimulation was performed with 0.5 mM **(A)** or 50 μM **(B and C)** ADPRP in the pipette solution. The intracellular Ca^2+^ concentration was adjusted to 1 μM. The respective current characteristics are indistinguishable from those evoked with ADPR as stimulus. **(D)** Comparison of the effects of different ADPRP concentrations on each channel variant. Note that 50 μM ADPRP failed to stimulate currents in *h*TRPM2, whereas in both *nv*TRPM2 variants significant responses were detected already at 5 μM. Asterisks indicate significant differences (* P < 0.05; ** P < 0.01, *** P < 0.001; Student's t-test, n = 5–9) in comparison to the absence of a stimulus. Error bars are s.e.

Along with ADPR, a further activator of *h*TRPM2 has been recently demonstrated [22]: Adenosine diphosphate ribose-2’-phosphate (ADPRP). This is confirmed in **Fig 5A** for *h*TRPM2. On *nv*TRPM2, ADPRP was effective as well (**Fig 5B**). Finally, ADPRP evoked currents in *nv*TRPM2-ΔNUD (with or without 3xHA-tag; **Fig 5C and 5D**) similarly as ADPR (**Fig 4D and 4E**). The concentration-effect-relations (**Fig 5D**) reveal that considerably high concentrations of ADPRP are required for the activation of *h*TRPM2, which are higher than they are known for stimulation with ADPR. This is in line with a report that ADPRP is a slightly weaker agonist on *h*TRPM2 than ADPR [22]. Highly potent effects of ADPRP were obtained for each of the *nv*TRPM2 variants (**Fig 5D**).

### Sensitivity of *nv*TRPM2 to H_2_O_2_ is induced by the deletion of the NUDT9H domain as well as by a C-terminal 3xHA-tag

**Fig 6:**
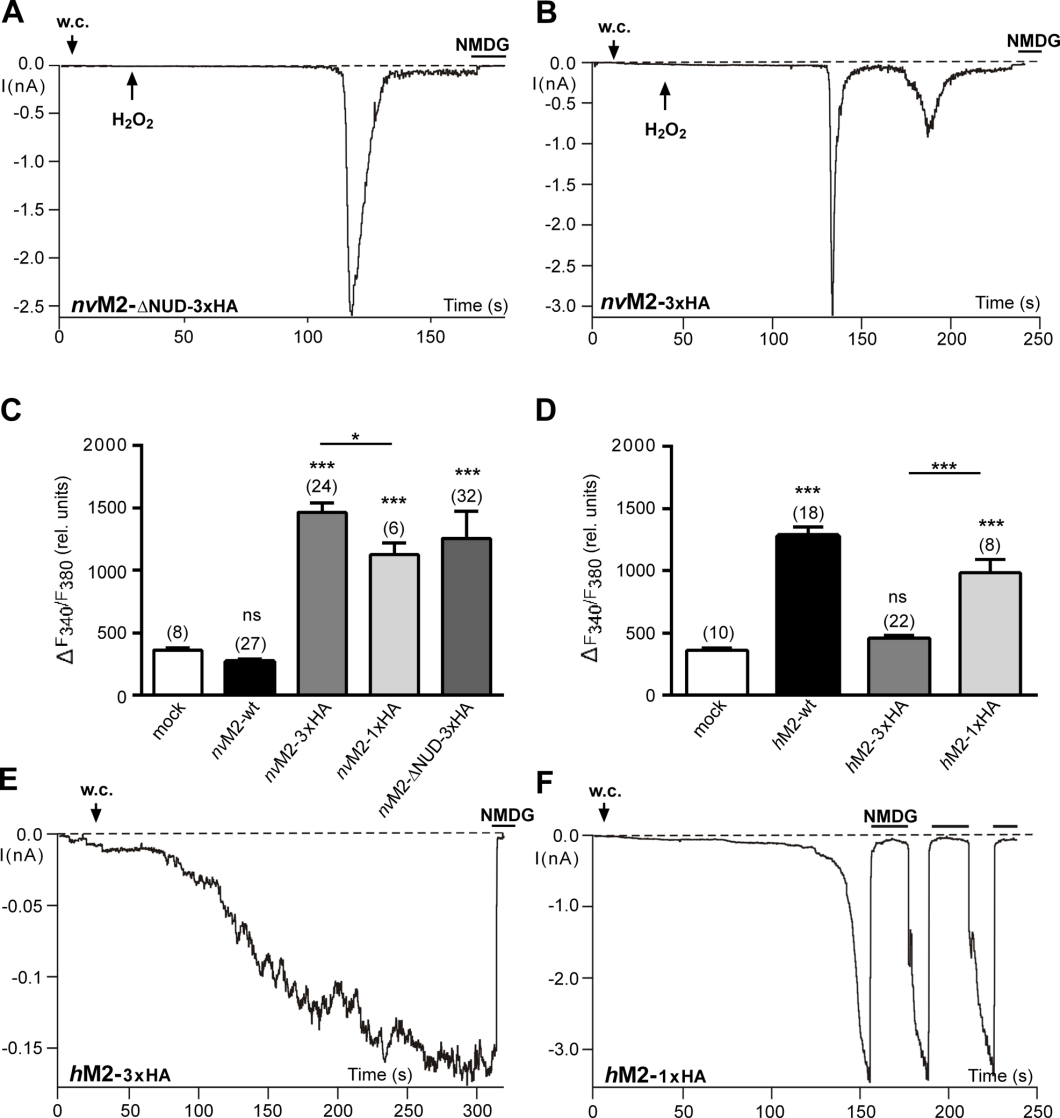
Functional effects of different C-terminally attached HA-tags on *h*TRPM2 and *nv*TRPM2 channel variants. **(A and B)** Characteristic currents of *nv*TRPM2- Δ NUD-3xHA **(A)** and *nv*TRPM2-3xHA **(B)** developed after the application of H_2_O_2_ (10 mM) to the bath (at the time point indicated by an arrow). The intracellular Ca^2+^ concentration was adjusted to 1 μM. Note that wild-type *nv*TRPM2 without an HA-tag is not stimulated by H_2_O_2_ (see Fig 2B inset). **(C and D)** Summary of the effects of extracellular H_2_O_2_ (10 mM) on several *nv*TRPM2 **(C)** and *h*TRPM2 **(D)** variants as obtained in calcium-imaging experiments. Note that the 3xHA-tag abolishes H_2_O_2_ sensitivity in *h*TRPM2 but creates it in *nv*TRPM2. **(E)** Attenuated current responses in *h*TRPM2-3xHA stimulated with ADPR (0.6 mM) and Ca^2+^ (1 μM) in the pipette solution. **(F)** After shortening of the 3xHA-tag, robust currents were evoked in *h*TRPM2-1xHA by ADPR (0.2 mM, n = 4). *** indicates a significant difference (P < 0.001) evaluated with a one-way ANOVA and the Bonferroni correction (n = 8–32). Error bars are s.e.

H_2_O_2_ responses of *nv*TRPM2 variants, already created by specific mutations within the NUDT9H region (ref. to **Fig 2**), were again evident after complete removal of this region. The *nv*TRPM2-ΔNUD variant (with or without 3xHA-tag) consistently exhibited currents after extracellular application of H_2_O_2_ (**Fig 6A**). Surprisingly, comparable H_2_O_2_-induced currents were even observed in *nv*TRPM2-3xHA (**Fig 6B**). The electrophysiological findings were confirmed in calcium imaging experiments (**Fig 6C**) where large and highly significant H_2_O_2_ responses were found in *nv*TRPM2-3xHA and *nv*TRPM2-ΔNUD-3xHA but never in untagged *nv*TRPM2. The H_2_O_2_-evoked small increases in (Ca^2+^)i in cells transfected with wild-type *nv*TRPM2 were unrelated to channel opening as they were also present in mock-transfected controls (**Fig 6C**). Thus, H_2_O_2_ sensitivity of *nv*TRPM2 was gained by alterations within the NUDT9H domain that most probably eliminate the enzymatic function, by the complete removal of the domain and furthermore by an inconspicuous manipulation near the C-terminus. The 3xHA-tag did also drastically affect *h*TRPM2 as calcium imaging experiments revealed almost abolished responses to H_2_O_2_ (**Fig 6D**). In the same line, electrophysiological experiments showed strongly reduced current amplitudes even in the presence of high intracellular concentrations of ADPR (n = 11; **Fig 6E**). Therefore, once again specific manipulations of the NUDT9 domain reduced channel function of *h*TRPM2, in striking contrast to the results obtained with *nv*TRPM2.

Since the sensitivity of *nv*TRPM2 and even *nv*TRPM2-ΔNUD to ADPR was not significantly impaired after the attachment of a C-terminal 3xHA-tag (ref. to **Fig 4C–4E**), the experimental data suggest that the function of the NUDT9H domain is specifically disturbed. We tried to reduce this negative impact by changing the 3xHA tag into an 1xHA-tag, both in *nv*TRPM2 and in *h*TRPM2. The results of these experiments are shown in **Fig 6C–6F**. Indeed, shortening of the 3xHA-tag restored channel function of *h*TRPM2 on the one hand (**Fig 6D and 6F**) and significantly reduced responses of *nv*TRPM2 to H_2_O_2_ on the other hand (**Fig 6C**). These data again indicate a different functional role of the NUDT9H domain in the two TRPM2 channel orthologs.

### Further experimental evidences for an enzymatic active role of the NUDT9 domain of *nv*TRPM2

**Fig 7:**
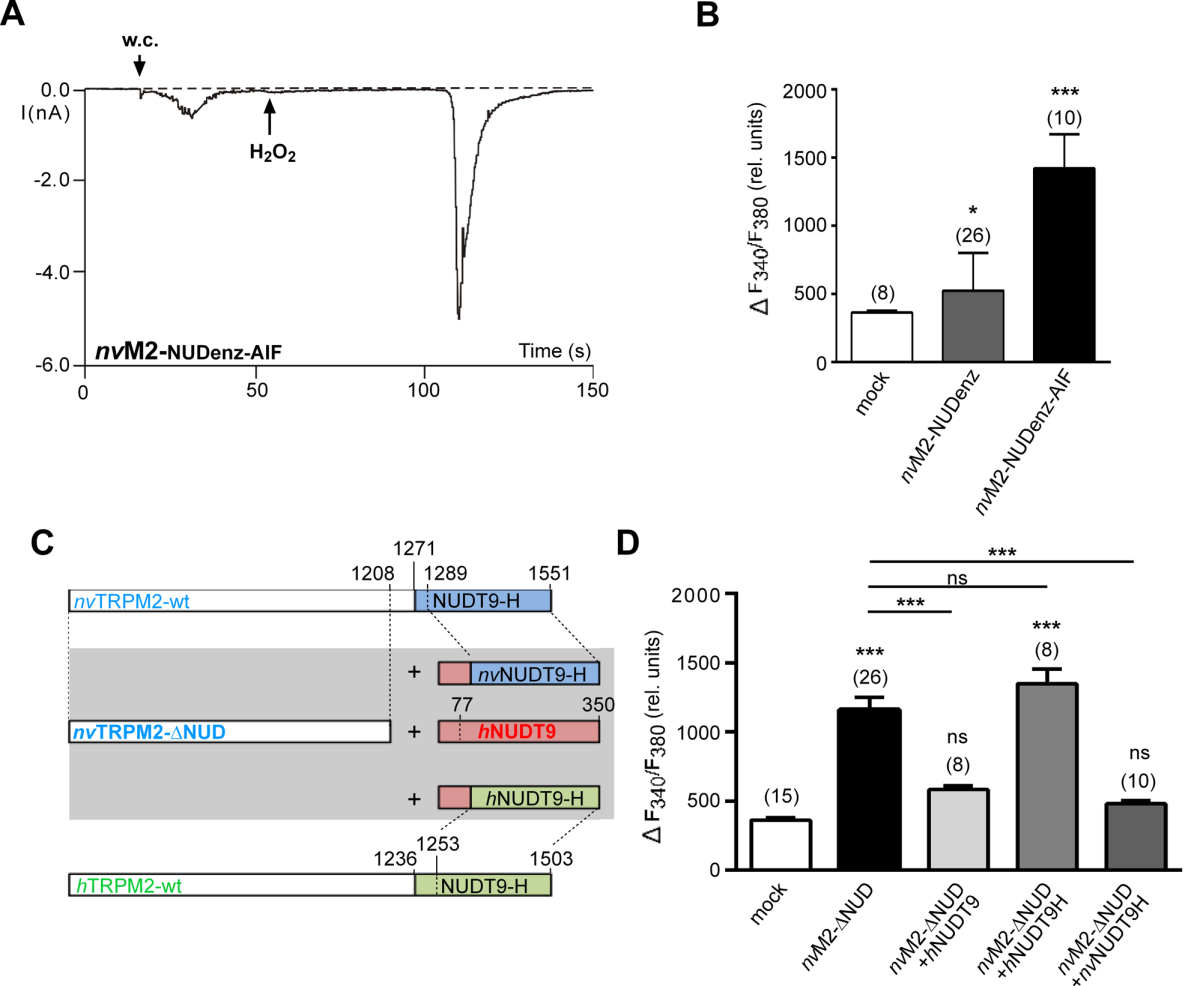
The sensitivity to H_2_O_2_ correlates with the catalytic activity of the NUDT9 domain. **(A)**, Representative whole cell patch-clamp experiment showing the stimulation of the variant *nv*TRPM2-NUDenz-AIF with H_2_O_2_ (10 mM) applied to the bath at the time point indicated by an arrow. The intracellular Ca^2+^ concentration was adjusted to 1 μM. Similar results were obtained from 6 independent experiments. **(B)** Summary of calcium imaging experiments. Maximal increases in (Ca^2+^)i, as indicated by an increased F340/F380 ratio, were evoked by extracellular application of H_2_O_2_ (10 mM). The variants *nv*TRPM2-NUDenz and *nv*TRPM2-NUDenz-AIF were compared with mock-transfected cells. **(C)** Sketch of the NUDT9 enzyme variants (wild-type *h*NUDT9 enzyme, *h*NUDT9H domain, *nv*NUDT9H domain) used for co-expression experiments with *nv*TRPM2-ΔNUD. **(D)** Calcium imaging experiments in response to H_2_O_2_ (10 mM), performed on cells co-expressing *nv*TRPM2-ΔNUD and one of the enzyme variants depicted in **panel C**. Comparison was performed with mock-transfected cells and cells transfected with *nv*TRPM2-ΔNUD alone. Asterisks indicate significant differences (* P < 0.05; *** P < 0.001) evaluated with a one-way ANOVA and the Bonferroni correction. (n = 8–26). Error bars are s.e.

So far the results demonstrate that the NUDT9 domain of *nv*TRPM2 is not directly involved in ADPR-dependent channel gating, whereas it has a crucial role in preventing responses to H_2_O_2_ which is regarded to induce intracellular accumulation of ADPR [16]. The comparison of critical sequences between the NUDT9H domain of *nv*TRPM2 and the native NUDT9 enzyme [5] strongly suggest that the domain in *nv*TRPM2 is enzymatically active. In the absence of a direct biochemical approach that could determine the catalytic activity of a channel domain, we analyzed the *nv*TRPM2 variant *nv*TRPM2-NUDenz where the endogenous NUDT9H domain was replaced by the corresponding sequence of the actual human NUDT9 enzyme. This variant was already described in our previous paper on *nv*TRPM2 and was found to be insensitive to H_2_O_2_ in whole-cell patch-clamp measurements [5]. We now changed the original sequence 1440-REF-1444 within the catalytic site of the NUDT9 domain in *nv*TRPM2-NUDenz to 1440-AIL-1444, a manipulation which almost completely abolished enzymatic function of the native NUDT9 enzyme [14]. As demonstrated in **Fig 7A** the *nv*TRPM2-NUDenz-AIF variant did indeed exhibit strong responses after extracellular stimulation with 10 mM H_2_O_2_, which was never observed in controls, i.e. *nv*TRPM2-NUDenz (n = 11, see also ref. 5). The electrophysiological results (**Fig 7A**; n = 6) were confirmed in calcium imaging studies (**Fig 7B**) where extracellular H_2_O_2_ induced large increases in (Ca^2+^)i in cells transfected with *nv*TRPM2-NUDenz-AIF. In control cells, either mock-transfected or transfected with *nv*TRPM2-NUDenz, no such increases in (Ca^2+^)i occurred. In slight deviation of the electrophysiological studies, the *nv*TRPM2-NUDenz variant showed minor but significant responses after stimulation with H_2_O_2_, in comparison to mock-transfected controls (**Fig 7B**).

To gain further evidences for an enzymatic activity of the NUDT9H domain of *nv*TRPM2, we performed co-expression experiments based on an approach already carried out in a previous study [16]. In particular, we co-expressed the *nv*TRPM2 channel lacking the NUDT9H domain (along with green fluorescence) together with one of the following NUDT9 variants: the native *h*NUDT9 enzyme, the NUDT9H domain of *nv*TRPM2, or the NUDT9H domain of *h*TRPM2. Each NUDT9 variant was expressed in a separate expression vector producing red fluorescence in HEK-293 cells (**Fig 7C**; ref. to Material and Methods). For the human NUDT9 enzyme, the successful co-expression was exemplarily confirmed with Western-blot analysis (**S1 Fig**). As demonstrated in calcium-imaging experiments (**Fig 7D**), the co-expression either of the native *h*NUDT9 enzyme or of the NUDT9H domain of *nv*TRPM2 significantly reduced the response of *nv*TRPM2-ΔNUD to H_2_O_2_, if compared to the positive controls (expression of *nv*TRPM2-ΔNUD alone). In order to exclude expression-related artifacts, we used the co-expression of the NUDT9H-domain of *h*TRPM2 as negative control because this domain is expected to be largely devoid of ADPRase activity [14–17]. Increases in (Ca^2+^)i were not suppressed by this NUDT9 variant (**Fig 7D**), suggesting that increased concentrations of ADPR after H_2_O_2_ application could not be reduced.

## Discussion

As a main finding, the present paper demonstrates gating of *nv*TRPM2 by ADPR independent of the NUDT9H domain. When the NUDT9H domain was modified or removed, the *nv*TRPM2 channels remained fully sensitive to ADPR and ADPRP, and were moreover activated by H_2_O_2_, an experimental model substance for oxidative stress which does not stimulate wild-type *nv*TRPM2 [5]. These findings necessitate a new interpretation of the role of the NUDT9H domain in *nv*TRPM2. In strict contrast to the situation in *h*TRPM2 where NUDT9H is decisive for channel activation by ADPR and by H_2_O_2_, its role in *nv*TRPM2 seems to be independent from channel gating and confined to the control of intracellular ADPR concentrations.

In *h*TRPM2, several modifications of the NUDT9H domain, in its putative ADPR-binding region as well as in its catalytic domain, have been described to completely abolish channel function [1;15;16]. Here we find that even the C-terminal attachment of a triple (3x) HA-tag profoundly compromises activation of *h*TRPM2. The HA-tag (especially in triplicate) represents a strong immuno-reactive epitope (a total of 27 residues with 6 aspartates and 6 prolines) but can occasionally interfere with protein function [23]. In contrast, it has been demonstrated in a previous study that a single (1x) FLAG-tag (8 hydrophilic, mostly acidic residues) fused to the C-terminus of the human TRPM2 channel has no functional impact [24]. This observation is in line with our experimental finding that the conversion of the 3xHA tag into a 1xHA tag largely restores the function of the *h*TRPM2 channel.

In *nv*TRPM2, NUDT9H seems to be similarly susceptible to already subtle modifications because many of these studied here and in our previous paper [5] created sensitivity to H_2_O_2_, or, as in our new interpretation, caused loss of downregulation of intracellular ADPR. Remarkably, this includes always mutations that presumably diminish enzymatic function of the NUDT9 enzyme [10].

Furthermore, from the data obtained with the channel variants containing a C-terminally fused HA-tag, it is concluded that this modification exclusively affects the function of the NUDT9H domain. Both in *nv*TRPM2-3xHA and in *nv*TRPM2-ΔNUD-3xHA, channel gating was not significantly changed. This finding supports our main point that channel gating of *nv*TRPM2 is independent of the NUDT9H domain. In contrast, *h*TRPM2-3xHA exhibits complete loss of channel function because of the presumed close interrelationship between channel and the NUDT9H domain.

Moreover, the present findings allow to understand some previous observations hard to explain at the time [5]. In particular, we substituted the native NUDT9H domain of *nv*TRPM2 with its counterpart of *h*TRPM2, yielding H_2_O_2_-sensitive channels without affecting the sensitivity to ADPR. However, when the corresponding region of the original human NUDT9 enzyme was used as substitute (variant *nv*TRPM2-NUDenz) the sensitivity to ADPR remained unaffected but no H_2_O_2_ effects were observed in patch-clamp measurements [5].

These data can now be taken as evidence for the essential role of enzymatic NUDT9H activity for the availability of intracellular ADPR for channel activation of *nv*TRPM2. This conclusion is further supported by two different experimental approaches. First, the variant *nv*TRPM2-NUDenz-AIF showed strong responses to H_2_O_2_, most likely as consequence of the neutralization of the catalytic site of the attached NUDT9 enzyme domain. Some residual sensitivity of *nv*TRPM2-NUDenz to H_2_O_2_ was detected exclusively in calcium imaging experiments and may reflect a strongly reduced but not abrogated catalytic activity in the artificial chimeric channel construct. Second, we performed co-expression experiments of *nv*TRPM2-ΔNUD with one of the following independent enzyme variants: *h*NUDT9 enzyme, *h*NUDT9H domain and *nv*NUDT9H domain and compared the responses to H_2_O_2_ in calcium imaging experiments. If the generally accepted hypothesis [16] is correct that H_2_O_2_ induces intracellular accumulation of ADPR, the catalytic activity of the used enzyme variants should be reflected by a reduced Ca^2+^ influx through *nv*TRPM2-ΔNUD. Then, the data clearly demonstrate that the ADPRase activity is similar in the NUDT9H domains of both, the *nv*TRPM2 channel and the *h*NUDT9 enzyme, but absent in the NUDT9H domain of *h*TRPM2.

The surprisingly different species-dependent properties of TRPM2 might provide valuable information for a better understanding of the gating mechanism of the human orthologue. Interestingly, the number of cysteine residues within both, the NUDT9 domain and the channel domain, is much higher in *h*TRPM2 than in *nv*TRPM2 as well as in the NUDT9 enzyme (**S2 Fig**). This again points to a role of NUDT9H in *nv*TRPM2 independent of channel gating and limited to enzymatic functions, whereas it is an interaction of NUDT9H with other parts of the channel that is likely to evoke gating in *h*TRPM2. Support for this idea derives from findings [25] that two conserved cysteine residues within the pore region of *h*TRPM2 are obligatory for channel function. These residues are missing in *nv*TRPM2.

**Fig 8:**
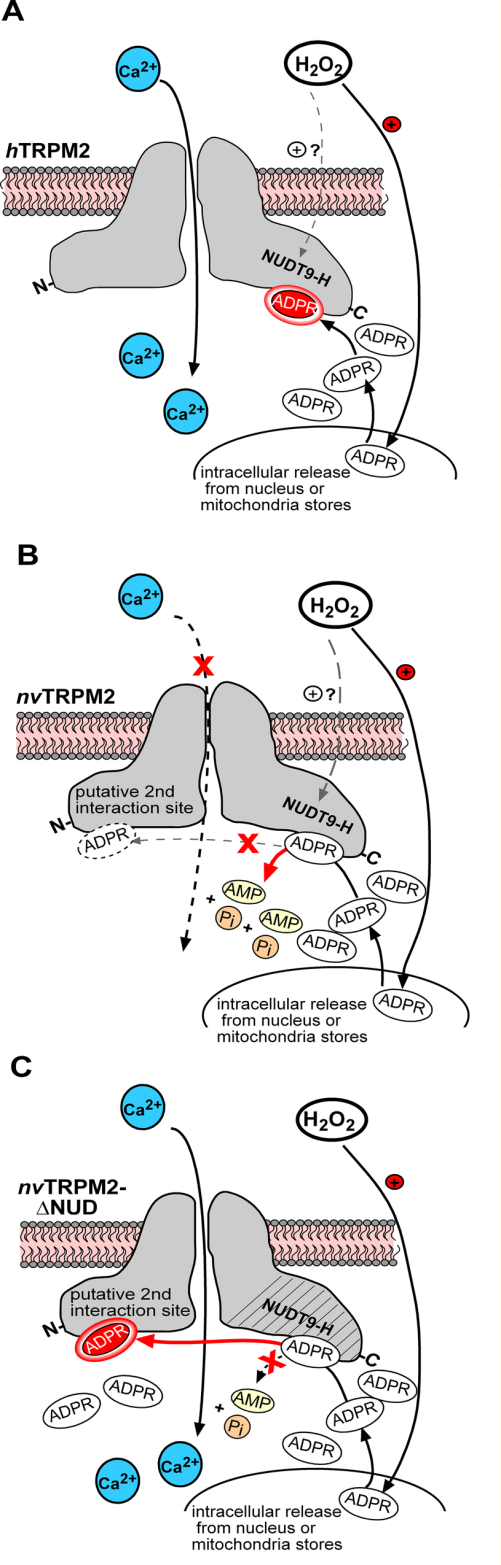
Sketch illustrating the different function of the NUDT9H domain in the two TRPM2 channel orthologs. **(A-C)** Cartoon interpretation of the putative functional role of the endogenous NUDT9H domain in heterologeously expressed *h*TRPM2, *nv*TRPM2 and *nv*TRPM2-ΔNUD as indicated during cell exposure to H_2_O_2_. It is generally accepted that oxidative stress leads to intracellular release of ADPR. In case of *h*TRPM2, the accumulated ADPR binds to the NUDT9H domain and initiates channel activation without the requirement of ADPRase activity **(A)**. In contrast, there is a second ADPR interaction site in *nv*TRPM2 responsible for gating, whereas the NUDT9H domain has a strictly enzymatic role, preventing channel activation by low cytosolic concentrations of ADPR as in the presence of H_2_O_2_. Hypothetically, H_2_O_2_ may additionally enhance the ADPRase activity of the NUDT9H domain **(B)**. When the catalytic activity of the NUDT9H domain of *nv*TRPM2 is lost due to point mutations or deletion of the entire domain, increasing concentrations of intracellular ADPR activate the channel **(C)**.

The proposed differences regarding the functional role of the NUDT9 domain in the TRPM2 orthologues of human and sea anemone are illustrated in **Fig 8**.

The present experiments in combination with previous ones [5] provide strong evidence that the regulatory function exerted by the NUDT9H domain of *nv*TRPM2 depends on ADPR pyrophosphatase activity. It is tempting to speculate that H_2_O_2_ leads to local or general accumulation of ADPR, deriving from enhanced activity of PARP and PARG enzymes [16;26;27] present also in *N*. *vectensis* [8;28]. This local ADPR does not necessarily lead to *nv*TRPM2 channel activity because it is successfully degraded by the channel itself. Hence, *nv*TRPM2 may truly represent a chanzyme, probably in contrast to its human orthologue for which no enzymatic activity is documented [17]. The idea of an autoregulated *nv*TRPM2 channel is not free of contradictions, however, because we have to ask how then activation of *nv*TRPM2 takes place principally and how the required ADPR or ADPRP concentrations are achieved if not by oxidative stress, as suggested [16]. Furthermore, *nv*TRPM2 variants without enzymatic activity should be more sensitive to ADPR than wild-type *nv*TRPM2, for which no evidence was obtained. In this context our observations may be relevant that the H_2_O_2_ sensitive variants of *nv*TRPM2 sometimes show spontaneous transient current responses immediately after reaching the whole-cell configuration and regularly produced spontaneous Ca^2+^-oscillations in calcium imaging experiments [5]. Such spontaneous events in ADPRase deficient but highly ADPR sensitive *nv*TRPM2 variants may be induced by basal levels of intracellular ADPR approaching a threshold. Naturally, these ADPR concentrations should be larger in intact cells (calcium imaging studies) than during infusion of the ADPR-free pipette solution in whole-cell patch clamp experiments.

Moreover, it should be taken into account that H_2_O_2_ is capable of regulating human NUDT9 pyrophosphatase activity [29]. In the presence of H_2_O_2_, the preferred activating divalent cation becomes Mn^2+^ rather than Mg^2+^ which can no longer act as cofactor; this is accompanied by an increased *K*_m_ for ADPR. Accordingly, treatment with H_2_O_2_ virtually abolishes enzymatic activity with Mg^2+^ as cofactor [29]. Such effects cannot directly explain the experimental data on *nv*TRPM2. However, since there is only a sequence homology of 49% between human NUDT9 enzyme and the NUDT9H domain of *nv*TRPM2, stimulating rather than inhibiting effects of H_2_O_2_ on pyrophosphatase activity might well be present.

The present findings on *nv*TRPM2 reveal a novel and previously unknown action of ADP-ribose. This action is completely different from that on the human TRPM2 orthologue, and does not seem to involve ADP-ribosylation either. While it is generally accepted that ADPR binds to the NUDT9H domain, no additional site is known in *nv*TRPM2 (and especially not for *nv*TRPM2-ΔNUD) that would enable an interaction with ADPR. A sequence comparison between channel domain and NUDT9H failed to detect the presence of another NUDIX-like region. Thus, a so far unknown mode of interaction between *nv*TRPM2 and ADPR must be assumed. However, already the fast on-kinetics of ADPR-induced currents in whole-cell studies would support the idea of a direct activation mechanism, without participation of enzymatic modification. Even stronger evidence is provided by the inside-out experiments where a prompt channel stimulation was consistently observed as soon as ADPR came into contact with the cytosolic side of the patch, in a cell free system.

Remarkably, traces from all three TRPM2 variants, *h*TRPM2, *nv*TRPM2, and *nv*TRPM2-ΔNUD looked very similar, not only in terms of channel slope conductance but particularly in terms of a quite unique feature of *h*TRPM2, the long open times interrupted by short flickering. If these properties of *h*TRPM2 are attributed to the gating process by the NUDT9H domain, it seems that the analogous mechanism in *nv*TRPM2 leads to similar behavior of the pore.

## Conclusion

Mammals as well as the far distantly related sea anemone have developed a Ca^2+^ permeable cation channel that is activated by intracellular ADPR. Thus, ADPR gating is very likely to represent a functionally relevant mechanism to deal with environmental challenges. In humans, a large body of evidence has accumulated that TRPM2 mediates a response to oxidative stress that may eventually result in apoptosis (e.g. reviewed in [30–33]). In *N*. *vectensis*, relatively little is known about analogous mechanisms but oxidative and thermal stress is certainly a relevant factor in the extremely variable habitat of this organism [34–37]. In spite of the fact that the ADPR-sensitive channels in either species are both TRPM2 orthologues, presumably evolving from a common ancestor already containing the NUDT9 domain [6], the intramolecular mechanism how ADPR evokes channel gating are surprisingly distinct. The same holds true for the role of the NUDT9H domain including its species-variable enzymatic activity. Thus, TRPM2 illustrates how one particular protein can be modified during divergent evolution to serve similar functions (ADPR-directed gating) by quite contrasting mechanisms, as well as to use the same basic structure (NUDT9H) for opposite responses to environmental challenges (oxidative stress).

## Supporting Information

S1 Fig Western-blot demonstrating successful co-expression of *nv*TRPM2-ΔNUD-3xHA and *h*NUDT9 enzyme. Western blot on total cell lysates of HEK-293 cells either transfected separately with *nv*TRPM2-ΔNUD-3xHA and *h*NUDT9 enzyme or co-transfected with both cDNAs (as indicated). Blotting membrane was divided (indicated by dashed line) and was probed with anti-HA antibody (upper part) or with monoclonal mouse anti-*h*NUDT9 antibody (lower part). Two independent experiments were performed to give similar results. The same transfection protocol was used for cells examined in calcium-imaging studies.
(TIF)

S2 Fig Distribution of cysteine residues in *h*TRPM2, *nv*TRPM2 and in the NUDT9 enzyme. The amino acid sequences are shown in FASTA format (single letter code). The putative transmembrane regions of *h*TRPM2 and *nv*TRPM2 are depicted in yellow and the NUDT9H domains of the channels are grayed. The cysteine residues within the sequences are highlighted in red letters. Note the relative accumulation of cysteine residues within the transmembrane regions as well as within the NUDT9H domain of human TRPM2.
(DOC)
