# Supplementary figures and images for: ADP-Ribose Activates the TRPM2 Channel from the Sea Anemone Nematostella vectensis Independently of the NUDT9H Domain
Source: PLoS One. 2016 Jun 22;11(6):e0158060. doi: 10.1371/journal.pone.0158060 (PMC4917252; doi:10.1371/journal.pone.0158060)

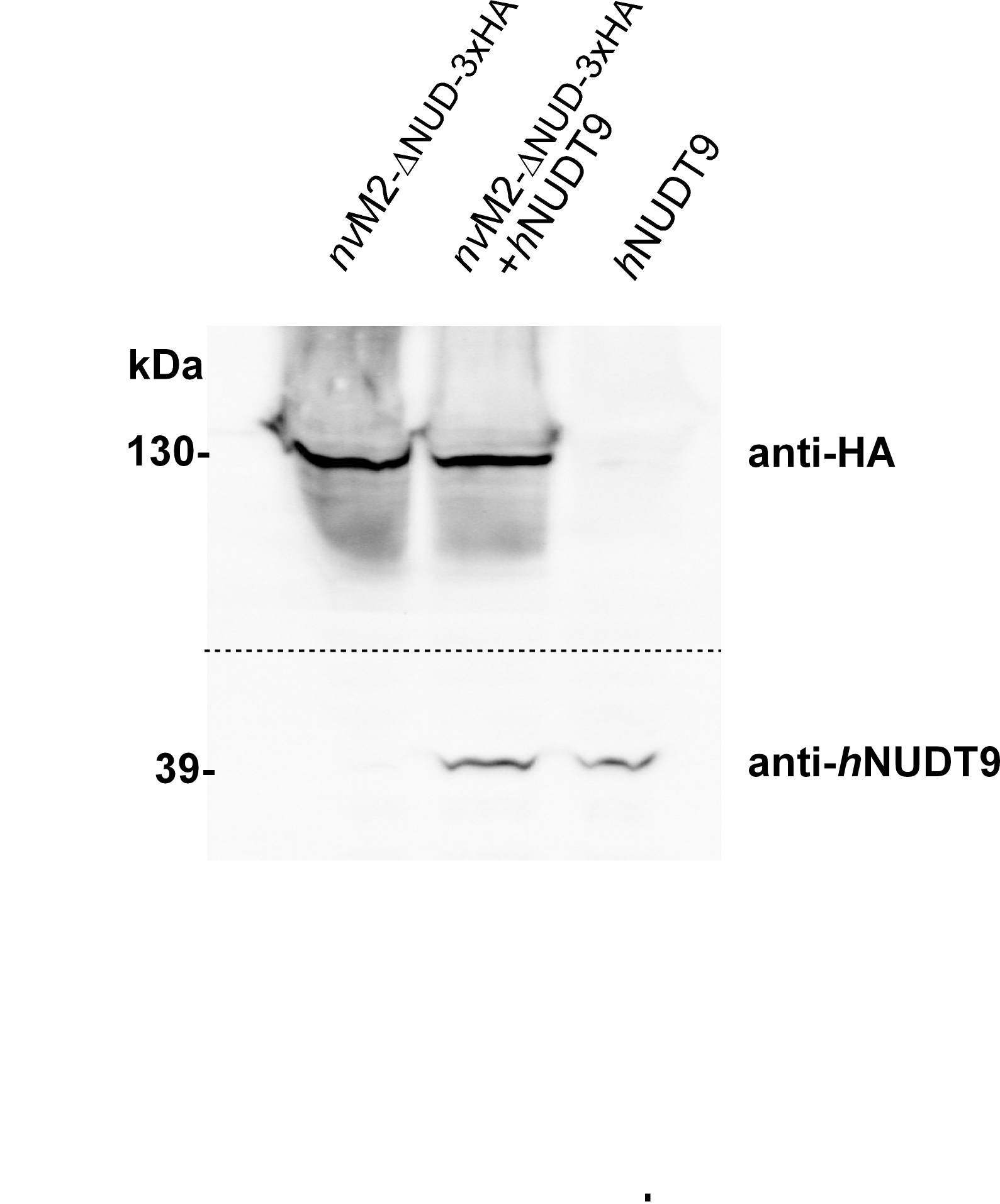

Supplement: S1 Fig — Western blot on total cell lysates of HEK-293 cells either transfected separately with nvTRPM2-ΔNUD-3xHA and hNUDT9 enzyme or co-transfected with both cDNAs (as indicated). Blotting membrane was divided (indicated by dashed line) and was probed with anti-HA antibody (upper part) or with monoclonal mouse anti-hNUDT9 antibody (lower part). Two independent experiments were performed to give similar results. The same transfection protocol was used for cells examined in calcium-imaging studies. (TIF) [file pone.0158060.s001.tif]
