# Supplementary material for: ADP-Ribose Activates the TRPM2 Channel from the Sea Anemone Nematostella vectensis Independently of the NUDT9H Domain
Source: PLoS One. 2016 Jun 22;11(6):e0158060. doi: 10.1371/journal.pone.0158060 (PMC4917252; doi:10.1371/journal.pone.0158060)
Supplement: S2 Fig — The amino acid sequences are shown in FASTA format (single letter code). The putative transmembrane regions of hTRPM2 and nvTRPM2 are depicted in yellow and the NUDT9H domains of the channels are grayed. The cysteine residues within the sequences are highlighted in red letters. Note the relative accumulation of cysteine residues within the transmembrane regions as well as within the NUDT9H domain of human TRPM2. (DOC) [file pone.0158060.s002.doc]

>sp|O94759|TRPM2_HUMAN

MEPSALRKAGSEQEEGFEGLPRRVTDLGMVSNLRRSNSSLFKSWRLQ**C**PFGNNDKQESLSSWIPENIKKKE**C**VYFVESSKLSDAGKVV**C**Q**C**GYTHEQHLEEATKPHTFQGTQWDPKKHVQEMPTDAFGDIVFTGLSQKVKKYVRVSQDTPSSVIYHLMTQHWGLDVPNLLISVTGGAKNFNMKPRLKSIFRRGLVKVAQTTGAWIITGGSHTGVMKQVGEAVRDFSLSSSYKEGELITIGVATWGTVHRREGLIHPTGSFPAEYILDEDGQGNLT**C**LDSNHSHFILVDDGTHGQYGVEIPLRTRLEKFISEQTKERGGVAIKIPIV**C**VVLEGGPGTLHTIDNATTNGTP**C**VVVEGSGRVADVIAQVANLPVSDITISLIQQKLSVFFQEMFETFTESRIVEWTKKIQDIVRRRQLLTVFREGKDGQQDVDVAILQALLKASRSQDHFGHENWDHQLKLAVAWNRVDIARSEIFMDEWQWKPSDLHPTMTAALISNKPEFVKLFLENGVQLKEFVTWDTLLYLYENLDPS**C**LFHSKLQKVLVEDPERPA**C**APAAPRLQMHHVAQVLRELLGDFTQPLYPRPRHNDRLRLLLPVPHVKLNVQGVSLRSLYKRSSGHVTFTMDPIRDLLIWAIVQNRRELAGIIWAQSQD**C**IAAALA**C**SKILKELSKEEEDTDSSEEMLALAEEYEHRAIGVFTE**C**YRKDEERAQKLLTRVSEAWGKTT**C**LQLALEAKDMKFVSHGGIQAFLTKVWWGQLSVDNGLWRVTL**C**MLAFPLLLTGLISFREKRLQDVGTPAARARAFFTAPVVVFHLNILSYFAFL**C**LFAYVLMVDFQPVPSW**C**E**C**AIYLWLFSLV**C**EEMRQLFYDPDE**C**GLMKKAALYFSDFWNKLDVGAILLFVAGLT**C**RLIPATLYPGRVILSLDFILF**C**LRLMHIFTISKTLGPKIIIVKRMMKDVFFFLFLLAVWVVSFGVAKQAILIHNERRVDWLFRGAVYHSYLTIFGQIPGYIDGVNFNPEH**C**SPNGTDPYKPK**C**PESDATQQRPAFPEWLTVLLL**C**LYLLFTNILLLNLLIAMFNYTFQQVQEHTDQIWKFQRHDLIEEYHGRPAAPPPFILLSHLQLFIKRVVLKTPAKRHKQLKNKLEKNEEAALLSWEIYLKENYLQNRQFQQKQRPEQKIEDISNKVDAMVDLLDLDPLKRSGSMEQRLASLEEQVAQTAQALHWIVRTLRASGFSSEADVPTLASQKAAEEPDAEPGGRKKTEEPGDSYHVNARHLLYPN**C**PVTRFPVPNEKVPWETEFLIYDPPFYTAERKDAAAMDPMGDTLEPLSTIQYNVVDGLRDRRSFHGPYTVQAGLPLNPMGRTGLRGRGSLS**C**FGPNHTLYPMVTRWRRNEDGAI**C**RKSIKKMLEVLVVKLPLSEHWALPGGSREPGEMLPRKLKRILRQEHWPSFENLLK**C**GMEVYKGYMDDPRNTDNAWIETVAVSVHFQDQNDVELNRLNSNLHA**C**DSGASIRWQVVDRRIPLYANHKTLLQKAAAEFGAHY

>jgi|Nemve1|248535|estExt_fgenesh1_pg.C_6220005

MGKDSFTPLYDGGDSSHVHLNKFGSNQLSQSKKSWIARNFSRRE**C**IRFVPKSHDVSR**C**K**C**GRPRERHSQQALESGQGSEEWNVAS**C**TTKHPTNAYGEIDFEGYGGQKRAPYLRMSHDTDANLVITLMLKRWNLEIPNLVISVTGGAKSFVLKPRLREMFRRGLIKAAKTTGAWIITGGTNTGVMKHVGEAVKEQQLMFGSDTQVNVIGIATWGIVDKQSDLISEKNGKYPALYSMEPTPGHQGAMLDPNHSHFFLVDDGTEGKYGVEIGMRSRIEEAIMKVKTDSRSEAGSIGVPVVLLVLEGGPNTVATMYELIKKKVPAVVIDGSGRAASVVGFAYNHTIKRNVDGQTINVIDPQYEDEVRAKVVEVFGAKGADKTYSMIKDVLEDEKMISVYSLDGEISQDIDLAILKALLKANRSSPVAQLNLALAWNRIDLAKSDIFTEEQQWTTETLSAAMLTALLDDKAEFAELFLQNGLSMREFLSLDIL**C**KLYAEVPGNTTIKPLLQKEMGKRQVKTIDMDVVGEVIEELMGDMFESYYRKDGHYFGELASYAEGLVLKNRKSSKDLLANINRIDPLPTPYLDVFLWAVL**C**NRRELARVLWEAGREPMAAALMASRLLKRMASRAQEDNTITDISSDLYDHARLFEERAVGVLDE**C**FNENETLSQTLLVRELDHYSRMTALELAVSAESQDFIAHTS**C**QVLLTRLWMGTMAMNTRWWKVLV**C**LYLPVLIFPIIYFVPDEQHERQAAEREHQKSLNQKSSKVKSHKEKNDAPVVPVYRSKEEKAVSNDEEARVGTENEEEDFQLEDYIPEIREDDSMEVIMRNKKLGF**C**DRIMHFYSAPFSKFVGNVVGYLAFIFLYAYVVLFNFPRFDPAKTLGGIHPTEIVLYFWVFTILIEEIRQLAAKPPKYIKDKVSVYFSDTWNFVDIFSLTVFIIAIILRFFTNSRIFTASRIILSLDIIFFIVRSLQIFSVNRLLGPKLVMIQKMMQDLAQFIIILAVFTIAYGIALHAVMFPSPGIYARNNTWVTITSVVQYPYWQMYGELFLDEIQGEKPKEFGEVDPDGRWLSPLLLAIYMVFTNILLLNLLIAIFNYTFERVQEDSDKVWKFQRYDLVQEYHSRPVFAPPLVLLGHILIFIRWVWRM**C**R**C**GHPPRGSTMKIGLSPAEMEQMDNWEFQAAEMYIHQQQQKNSGTLEERVRALGDRVD**C**INSQLNRVLDSMSGTRAHALTDGNGLEGGHDSEGRLARMEVELSSNSESLQKILALLQQQPPVKGQAAVPIQLTLLHYKARSSPYPGSTAKRFAVQDNMVDWQVPFPDYKPVNYTAPVVLANPVWADKDLMAMSPRPELPYNQMDHT**C**NVNRVSYNGTYVVKDGLPLNPMGRTGMQGRGLLGRFGPNHAADPVVTRWKRTSAGVMLQGGKKVLEFVAIQRKDNNQWAIPGGMVEPGQLVTQALKAEFGEEAMAKLNVSQEEKERIAKQIERLFQQGQEIYKGYVDDPRNTDNAWMETVAVNFHDDKGDLFGDITLQAGDDAAAVRWQRVSGNIPLYASHVSILEKVAKMRDAAF

>sp|Q9BW91|NUDT9_HUMAN ADP-ribose pyrophosphatase, mitochondrial OS=Homo sapiens GN=NUDT9 PE=1 SV=1

MAGRLLGKALAAVSLSLALASVTIRSSR**C**RGIQAFRNSFSSSWFHLNTNVMSGSNGSKENSHNKARTSPYPGSKVERSQVPNEKVGWLVEWQDYKPVEYTAVSVLAGPRWADPQISESNFSPKFNEKDGHVERKSKNGLYEIENGRPRNPAGRTGLVGRGLLGRWGPNHAADPIITRWKRDSSGNKIMHPVSGKHILQFVAIKRKD**C**GEWAIPGGMVDPGEKISATLKREFGEEALNSLQKTSAEKREIEEKLHKLFSQDHLVIYKGYVDDPRNTDNAWMETEAVNYHDETGEIMDNLMLEAGDDAGKVKWVDINDKLKLYASHSQFIKLVAEKRDAHWSEDSEAD**C**HAL
